# Ananalysis of the effects of Treg cell therapy intervention on the gut microbiota of type 1 diabetic mice using 16S rRNA gene sequencing

**DOI:** 10.3389/ebm.2026.10701

**Published:** 2026-03-03

**Authors:** Mengyao Zhou, Kang Du, Hanmin Wang, Zhuxian Zhang, Rui Zhao, Chenghong Ma, Qionger Huang, Wei Zhang, Weiwen Chen

**Affiliations:** 1 Department of Endocrinology and Metabolism, Yunnan Qujing Central Hospital, Qujing, Yunnan, China; 2 Department of Neurology, Yunnan Qujing Central Hospital, Qujing, Yunnan, China

**Keywords:** 16S rRNA gene sequencing, ABC transporters, gut microbiota, T1DM, Treg

## Abstract

This study established a type 1 diabetes (T1DM) mouse model via intraperitoneal injection of streptozotocin (STZ) and examined the effect of regulatory T (Treg) cells on the gut microbiota by comparing its composition and diversity across three groups: control, T1DM, and Treg-treated mice. Forty-one 8-week-old male C57BL/6 mice under specific pathogen-free conditions were divided into a healthy control group, an untreated T1DM group, and a Treg treatment group (receiving low, medium, or high doses). T1DM was induced by administering a low-dose STZ injection over five consecutive days, with diabetes confirmation defined as a blood glucose level ≥300 mg/dL. CD4+CD25+ Treg cells isolated from spleens of healthy mice were used for treatment. Fecal samples collected on days 0, 14, and 34 from three randomly selected mice per group were subjected to 16S rRNA gene sequencing targeting the V3-V4 regions. The results showed significant differences in both alpha and beta diversity among the groups. Dominant bacterial families varied: Ruminococcaceae and others were enriched in the Treg treatment group, Muribaculaceae in the control group, and Lactobacillaceae in the untreated T1DM group. Genus-level abundances also shifted over time. Firmicutes abundance positively correlated with Treg levels (r = 0.70, p = 0.0433) but negatively with IFN-γ, whereas Cyanobacteria exhibited the opposite correlation. The Firmicutes/Bacteroidetes ratio was higher in T1DM mice than in controls and lower in the Treg-treated group. Metabolic pathway analysis indicated that two-component systems and ABC transporters were more prevalent in T1DM mice. In summary, Treg cell treatment altered the diversity, composition, dominant taxa, and Firmicutes/Bacteroidetes ratio of the gut microbiota compared with untreated T1DM mice.

## Graphical Abstract

**Figure:**
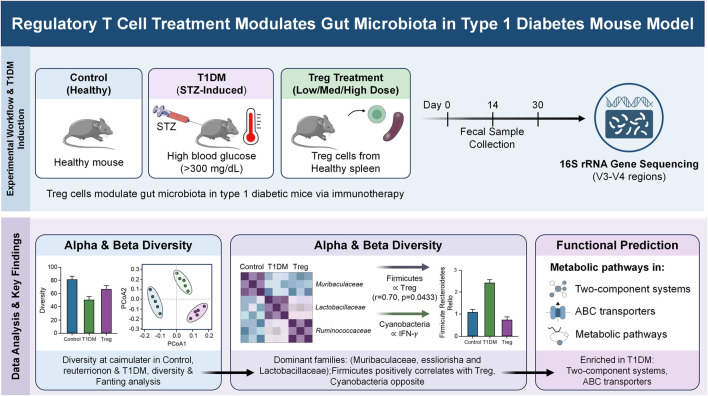

## Impact statement

This study is crucial for the field as T1DM remains a significant health challenge with limited treatment options. By exploring the role of Treg cells in modulating gut microbiota in T1DM, it advances the field by filling a knowledge gap. New information includes specific correlations between bacteria, Treg cells, and immune factors, and changes in gut microbiota profiles with Treg cell treatment. This impacts the field by offering potential novel therapeutic targets and a better understanding of T1DM pathogenesis, potentially leading to improved treatment strategies.

## Introduction

Type 1 diabetes mellitus (T1DM) is characterized by dysfunction and death of beta cells caused by reactive T cells, leading to abnormal synthesis and secretion of insulin. This results in glucose metabolism disorder characterized by absolute insulin deficiency and high blood sugar in patients [1]. The exact mechanism of the pathophysiology of T1DM is still unclear. Its etiology involves multiple factors, with the main known ones currently including autoimmunity, genetic susceptibility, viral infections, diet, and gut microbiota [2]. Advanced maternal age, infant obesit, premature intake of milk, high-sugar low-fiber diet are associated with an increased incidence of T1DM, and can also affect gut microbiota. With the deepening research on gut microbiota in recent years, it is suggested that gut microbiota may be a common pathway for multiple factors influencing the incidence of T1DM [3]. Treg cells in helper T cells have regulatory and anti-inflammatory functions, which can inhibit abnormal infiltration of CD8^+^ T cells in the pancreas and kidneys, thereby delaying the development of diabetes [4]. Through previous research, we have found that Treg cells can improve fasting blood glucose, body weight, 24-h urine volume, water intake, urine protein, as well as insulin and C-peptide levels in Streptozotocin-induced T1DM mice to a certain extent. They also have a role in improving renal inflammatory lesions in diabetic nephropathy, confirming the effectiveness of Treg cells in treating type 1 diabetes [5]. Existing research has confirmed that changes in the gut microbiota can induce or inhibit autoimmune responses in the pancreas, thereby affecting the occurrence of T1DM(5). Therefore, the relationship between intestinal flora and Treg cells may provide a new perspective for studying the pathogenesis and treatment of T1DM.

This study utilized intraperitoneal injection of Streptozotocin (STZ) to prepare T1DM mouse models, collected fecal samples from control group, T1DM group, and Treg cell treatment group for 16S rRNA sequencing, investigated the composition and function of gut microbiota in T1DM model mice, analyzed the impact of Treg cells on gut microbiota in T1DM mice, and further explored the potential mechanisms by which Treg cells exert their effects on the gut microbiota pathway in T1DM mice.

## Materials and methods

### The production of the T1DM model as well as the sorting and *in vitro* culture of CD4^+^CD25^+^Treg cells

A total of 41 male C57BL/6 mice were housed and allocated into three main groups: a healthy control group, an untreated T1DM group, and a Treg cell treatment group. The treatment group was further divided into three groups based on different doses of Treg cells (1 × 10^6^/kg, 5 × 10^6^/kg, 10 × 10^6^/kg). The T1DM model was induced by administering multiple low-dose intraperitoneal injections of streptozotocin (STZ; Sigma-Aldrich, St Louis, MO, USA). STZ was dissolved in 0.1 M citrate buffer (pH 4.5) at a concentration of 60 mg/kg and injected for five consecutive days. Blood glucose levels were monitored using an Embrace glucometer (OMNIS Health, USA) with compatible test strips. Successful diabetes induction was confirmed when blood glucose levels measured ≥300 mg/dL on two consecutive readings. For Treg cell preparation, the spleen was aseptically collected from a euthanized healthy C57BL/6 mouse. After removal of connective tissue and fat, the spleen was mechanically dissociated into a single-cell suspension. The suspension was passed through a cell strainer to remove debris and aggregates, then centrifuged. The supernatant was discarded, and red blood cells were lysed using a commercial lysis buffer. Following another centrifugation step, the pellet was resuspended in PBS. CD4^+^CD25^+^ Treg cells were subsequently isolated from this suspension using magnetic-activated cell sorting (MACS) and expanded via *ex vivo* culture prior to administration.

### Observation indicators, collection of stool samples, and grouping situations

On Day 1, mice received tail vein injections of either PBS buffer (control) or ex vivo-expanded Treg cells. Fasting blood glucose, body weight, 24-h water intake, and 24-h urine output were monitored on days 0, 4, 7, 10, 14, 18, 25, 32, and 34. Serum levels of insulin and C-peptide were measured using ELISA. Peripheral blood mononuclear cells (PBMCs) were analyzed by flow cytometry to determine the proportions of Th1, Th2, Th17, and Treg cells among splenic lymphocytes. Intracellular cytokine staining (ICS) coupled with flow cytometry was employed to measure the proportions of IFN-γ^+^, IL-4^+^, IL-17^+^, and Treg cells. Adverse events and complications were documented throughout the study. The therapeutic efficacy of Treg cell administration in treating T1DM was subsequently evaluated.

Fecal sample collection: Based on preliminary study findings, fecal samples were collected from three randomly selected mice per group immediately prior to euthanasia on days 0, 14, and 34. To collect fresh feces, mice were gently restrained, their tails were raised, and the lower abdomen was lightly pressed to stimulate defecation. Approximately 2–3 fecal pellets per mouse were collected directly into sterile microcentrifuge tubes. All samples were immediately frozen at −80 °C and subsequently transported on dry ice for further analysis.

At specific time points, the grouping of mice for euthanization is as follows: Day 0 (2 groups, each with 3 mice): Control (Day0-Con), T1DM (Day0-DM). Day 14 (5 groups, each with 3 mice): Control (Day14-Con), T1DM (Day14-DM), T1DM + Treg (Day14-Low-Treg, Day4-Med-Treg, Day14-High-Treg). Day 30 (5 groups, each with 3 mice): Control (Day30-Con), T1DM (Day30-DM), T1DM + Treg (Day30-Low-Treg, Day30-Med-Treg, Day30-High-Treg).

### 16S rRNA gene sequencing

Total genomic DNA was extracted from the mouse fecal samples. The V3-V4 hypervariable region of the bacterial 16S rRNA gene was subsequently amplified by PCR and sequenced on the Illumina platform (PE250) [6, 7]. For bioinformatic analysis, paired-end reads were first assigned to respective samples based on their unique barcodes and primers. After quality filtering, forward and reverse reads were merged using overlap sequences. The assembled sequences were then subjected to chimera removal. Operational Taxonomic Units (OTUs) were clustered from the quality-filtered sequences using UPARSE (version 7.1) with a 97% similarity threshold. OTUs with a relative abundance <0.001% across all samples were excluded to reduce noise. Chimeric sequences were further identified and filtered out using the UCHIME algorithm. Taxonomic classification of each 16S rRNA gene sequence was performed using the UCLUST algorithm against a reference database with a confidence threshold of 80%.

### Statistical analysis

Alpha diversity, reflecting the richness and diversity of the microbial community within each sample, was assessed by calculating the Observed species index, Chao1 index, Simpson index, and Shannon index using mothur software (version v.1.30.1). Beta diversity was evaluated through Principal Coordinate Analysis (PCoA), Principal Component Analysis (PCA), and Non-metric Multidimensional Scaling (NMDS), performed in R with the vegan package. Linear discriminant analysis Effect Size (LEfSe) was employed to identify bacterial taxa exhibiting significant differences between groups, using a linear discriminant analysis (LDA) score threshold >2.

Statistical analyses were conducted using GraphPad Prism (version 9.0; GraphPad Software, San Diego, CA, United States). Normally distributed parametric data were analyzed by one-way ANOVA, followed by Brown-Forsythe and Welch’s post-hoc tests for multiple comparisons. Non-parametric data were analyzed using the Kruskal-Wallis test. Statistical significance was determined at *p* < 0.0500.

## Results

### 16S rRNA sequencing results

**FIGURE 1:**
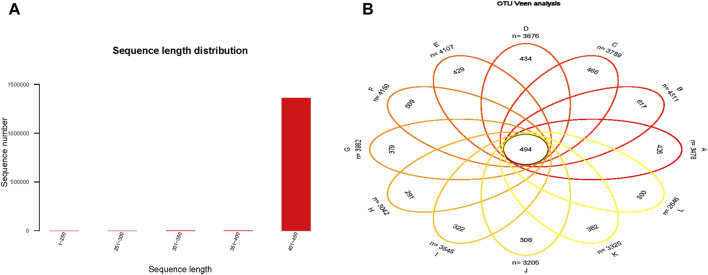
The sequences lengths distribution **(A)** and the Veen analysis **(B)**. **(A)** 99.85% of the sequences had lengths distributed within the range of 401–450 base pairs. **(B)** There were 494 OTUs shared among all groups.

Through 16S rRNA gene sequencing of fecal samples from 36 mice, 1,362,799 high-quality sequences were obtained, totaling 568,163,285 base pairs. Of these, 99.85% of sequences had lengths between 401 and 450 bp (Figure 1A), with a mean length of 416.91 bp, which falls within the expected range (400–440 bp) for the V3–V4 region.

Operational taxonomic units (OTUs) were clustered at 97% sequence similarity from all high-quality reads, yielding 12,015 OTUs after rarefaction. Following rarefaction, taxonomic assignment identified 1 domain, 32 phyla, 69 classes, 137 orders, 167 families, 292 genera, 289 species, and 11,948 OTUs. The OTU counts across groups were 3,478, 4,311, 3,789, 3,876, 4,107, 4,150, 3,982, 3,042, 3,545, 3,205, 3,325, and 2,646, with 494 OTUs shared among all groups. Statistically significant differences in OTU numbers were observed between group A (Day0-Con) and B (Day 0-DM) (*p* < 0.0001) and between group D (Day 14-DM) and F (Day 14-Med-Treg) (*p* = 0.0002) (Figure 1B).

### Analysis of community structure of different groups at different classification levels

**FIGURE 2:**
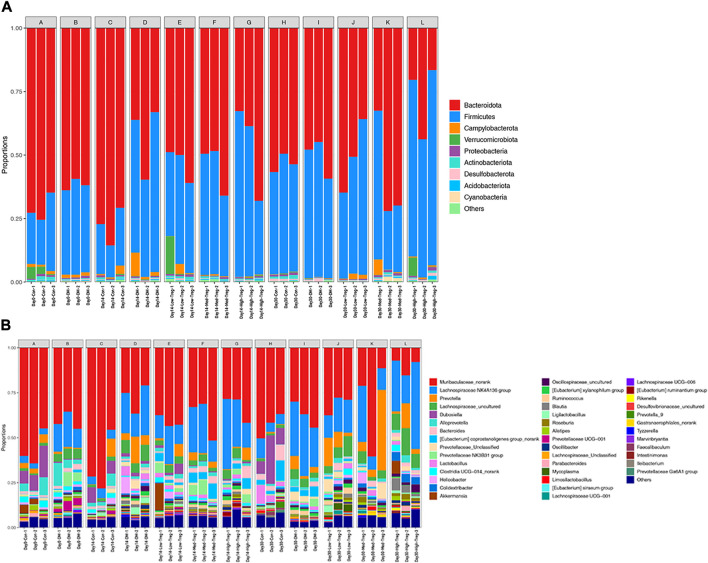
**(A)** Microbial community barplot of phylum. **(B)** Microbial community barplot of genus.

**FIGURE 3:**
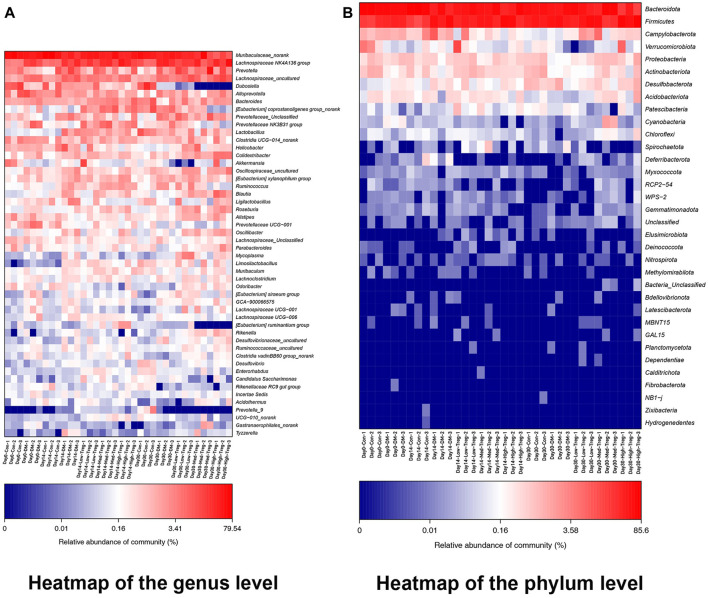
**(A)** The heatmap of the genus level. **(B)** The heatmap of the phylum level.

At the horizontal level of the phyla (Figure 2A), it is mainly composed of Bacteroidota (19.43%), Firmicutes (14.98%), Campylobacterota (0.42%), and Verrucomicrobiota (0.34%), with the average relative abundance of the remaining phyla all less than 0.3%. Combining this with the heatmap (Figures 3A,B), we found that Verrucomicrobiota showed a significant difference between groups A (Day 0-Con) and B (Day 0-DM), with higher abundance in group A, indicating a possible association between the reduction of Verrucomicrobiota and T1DM.

Notably, the Firmicutes/Bacteroidetes (F/B) ratio—an indicator linked to energy metabolism—was elevated in the T1DM mouse group compared to the control group at the detected time points, suggesting gut microbiota dysbiosis in T1DM mice. In contrast, the Tregs cell treatment group showed a reduced F/B ratio relative to the T1DM group, which was accompanied by a decrease in body weight, implying a potential role of this ratio in the therapy’s weight-modulating effect.

At Day 14, the average abundance of Bacteroidota, Actinobacteriota, and Acidobacteriota in the Treg cell treatment group was higher than in the diseased group. Among them, Actinobacteriota and Acidobacteriota had the highest abundance in the low-dose group, with a decreasing trend as the concentration increased. Campylobacterota had a higher abundance in the diseased group compared to the normal and treatment groups. By Day 30, Proteobacteria, Acidobacteriota, and Actinobacteriota in the Treg cell treatment group and normal group had higher abundances than in the diseased group (all p < 0.0500). Additionally, Proteobacteria and Actinobacteriota had relatively higher abundances in the medium-dose group, while Acidobacteriota showed an increasing trend with increasing concentration (Table 1).

**TABLE 1:** Relative abundance of each phylum in experimental groups at different time points.

Phylum	Day 0			Day 14							
	Control	T1DM	*p* value	Control	T1DM	Low-treg	Med-treg	High-treg	*p* value	*p* (C,D)	*p* (D,F)
Bacteroidota	21,039 ± 1,645	18,284 ± 664.6	0.1	23,067 ± 2,199	12,738 ± 4,309	15,787 ± 1986	169,188 ± 291	13,762 ± 5,600	0.0676	0.1	0.4
Firmicutes	6,783 ± 2081	10,392 ± 696.3	0.1	5,426 ± 1,540	15,157 ± 3,661	11,003 ± 1,520	12,632 ± 2,804	15,162 ± 5,742	0.0518	0.1	0.4
Campylobacterota	313.0 ± 72.17	357.7 ± 41.63	0.4	427.7 ± 472.3	1,151 ± 1,376	424.3 ± 633.1	107.0 ± 56.51	65.33 ± 43.55	0.1339	0.4	0.1
Verrucomicrobiota	782.0 ± 625.6	22.67 ± 7.095	0.1	56.00 ± 67.36	50.00 ± 28.16	1,522 ± 2,497	23.67 ± 6.658	67.00 ± 48.77	0.4708	>0.999	0.4
Proteobacteria	196.7 ± 83.68	177.0 ± 78.82	>0.999	170.3 ± 67.88	122.7 ± 63.72	218.0 ± 48.66	120.7 ± 82.95	149.3 ± 36.23	0.3056	0.7	>0.999
Actinobacteriota	309.7 ± 106.1	81.67 ± 27.68	0.1	181.0 ± 89.52	65.00 ± 28.79	246.0 ± 73.26	177.0 ± 52.12	123.7 ± 6.807	0.0436	0.3	0.1
Desulfobacterota	23.33 ± 8.083	74.00 ± 32.08	0.1	46.67 ± 26.96	132.0 ± 41.04	99.00 ± 102.9	52.67 ± 24.34	92.67 ± 40.10	0.1968	0.1	0.1
Acidobacteriota	96.33 ± 60.72	102.7 ± 30.09	0.8	91.33 ± 35.80	83.33 ± 70.87	107.0 ± 36.39	105.3 ± 8.505	87.33 ± 79.11	0.9703	>0.999	>0.999
Cyanobacteria	8.333 ± 2.517	65.00 ± 20.22	0.1	10.00 ± 6.245	17.33 ± 7.234	8.000 ± 3.606	8.000 ± 3.464	3.667 ± 6.351	0.3134	0.2	0.2

Phylum	Day 30							
	Control	T1DM	Low-treg	Med-treg	High-treg	*p* value	*p* (Con,DM)	*p* (Con,Med-treg)
Bacteroidota	15,785 ± 1,075	15,015 ± 2,251	14,944 ± 4,285	17,229 ± 6,580	7,974 ± 4,364	0.2203	0.7	0.7
Firmicutes	12,804 ± 1,054	14,119 ± 2,189	13,941 ± 4,065	10,654 ± 5,819	19,832 ± 3,385	0.1968	0.7	0.7
Campylobacterota	29.33 ± 9.074	48.33 ± 27.74	400.0 ± 289.0	649.0 ± 1,011	163.7 ± 24.44	0.0748	0.4	0.7
Verrucomicrobiota	69.00 ± 40.95	28.00 ± 30.79	1.667 ± 1.528	26.00 ± 6.00	728.7 ± 1,207	0.0837	0.2	0.8
Proteobacteria	227.7 ± 49.10	46.00 ± 6.083	104.3 ± 32.62	253.3 ± 18.18	219.7 ± 168.7	0.0133	0.1	0.1
Actinobacteriota	330.3 ± 99.29	57.33 ± 16.20	47.33 ± 2.887	162.0 ± 81.18	133.3 ± 117.6	0.0278	0.1	0.1
Desulfobacterota	237.7 ± 76.35	203.0 ± 183.8	89.33 ± 34.08	108.3 ± 80.83	230.0 ± 104.8	0.3705	>0.9999	>0.9999
Acidobacteriota	33.33 ± 28.45	13.33 ± 3.786	18.33 ± 9.866	135.7 ± 31.79	176.0 ± 228.5	0.0437	0.4	0.1
Cyanobacteria	7.667 ± 3.055	3.333 ± 2.082	14.67 ± 8.327	289.7 ± 258.5	7.000 ± 6.245	0.0532	0.2	0.1

Data are presented as mean ± standard deviation, summarizing the relative abundance of each phylum in control group, T1DM, group, and Treg intervention groups (low/medium/high dose) at Day 0, Day 14, and Day 30. For the data of Day14 and Day30, we used the Kruskal-Wallis test. All continuous variables are presented in the form of mean ± standard deviation. *p* values indicate inter-group difference significance (*p* < 0.0500).

At the genus level, the main bacteria are Muribaculaceae_norank (13.67%), Lachnospiraceae NK4A136 group (6.22%), Prevotella (1.85%), Lachnospiraceae_uncultured (1.60%), and Dubosiella (1.12%). The relative abundance of the remaining genera is less than 1.0% on average (Figure 2B). Importantly, two-genera comparisons (Tables 2, 3) and heatmaps (Figures 3A,B) identified specific taxa with marked changes. At Day 0, the abundance of the [Eubacterium] siraeum group was significantly higher in the T1DM group than in the normal control, suggesting a close relationship between this genus and the disease. Furthermore, the Day 14 comparison between the untreated T1DM and Treg-treated groups revealed significant differences in the abundances of *Helicobacter*, Tyzzerella, *Mycoplasma*, and the [Eubacterium] ruminantium group, pointing to their potential roles in the therapeutic response.

**TABLE 2:** Relative abundance ratios and corresponding taxonomic information of gut microbiota between control and T1DM groups at Day 0.

OTU	Day0-Con/DM	Taxonomy
OTU1035	77	p__Bacteroidota; g__norank
OTU625	64	p__Firmicutes; g__Dubosiella
OTU40	54.27777778	p__Verrucomicrobiota; g__Akkermansia
OTU1707	44	p__Bacteroidota; g__norank
OTU189	43.06666667	p__Actinobacteriota; g__Bifidobacterium
OTU422	40.75	p__Bacteroidota; g__Odoribacter
OTU1158	37.5	p__Bacteroidota; g__norank
OTU1726	37	p__Bacteroidota; g__norank
OTU31	36.84375	p__Verrucomicrobiota; g__Akkermansia
OTU226	36	p__Firmicutes; g__Faecalibaculum
OTU148	0.014218009	p__Bacteroidota; g__norank
OTU120	0.014064698	p__Bacteroidota; g__*Bacteroides*
OTU414	0.013157895	p__Bacteroidota; g__norank
OTU278	0.012345679	p__Firmicutes; g__[Eubacterium] siraeum group
OTU281	0.009433962	p__Firmicutes; g__[Eubacterium] siraeum group
OTU325	0.007874016	p__Firmicutes; g__[Eubacterium] siraeum group
OTU182	0.0078125	p__Bacteroidota; g__norank
OTU241	0.007352941	p__Bacteroidota; g__norank
OTU275	0.003745318	p__Firmicutes; g__Lachnospiraceae NK4A136 group
OTU194	0.002923977	p__Firmicutes; g__Lachnospiraceae NK4A136 group

**TABLE 3:** Relative abundance ratios and corresponding taxonomic information of gut microbiota between T1DM and Medium-dose Treg groups at Day 14.

OTU	Day14-Con/DM	Taxonomy
OTU67	537	p__Firmicutes; g__Lachnospiraceae NK4A136 group
OTU96	131	p__Firmicutes; g__Lachnospiraceae NK4A136 group
OTU81	130	p__Campylobacterota; g__*Helicobacter*
OTU186	109.6	p__Campylobacterota; g__*Helicobacter*
OTU285	104.5	p__Firmicutes; g__*Mycoplasma*
OTU268	93.66666667	p__Firmicutes; g__Lachnospiraceae NK4A136 group
OTU100	85.07692308	p__Campylobacterota; g__*Helicobacter*
OTU764	85	p__Firmicutes; g__uncultured
OTU232	81.75	p__Firmicutes; g__Tyzzerella
OTU263	80	p__Firmicutes; g__uncultured
OTU635	0.018181818	p__Bacteroidota; g__norank
OTU378	0.015384615	p__Bacteroidota; g__norank
OTU142	0.011494253	p__Firmicutes; g__Lachnospiraceae NK4A136 group
OTU393	0.011278195	p__Firmicutes; g__norank
OTU349	0.01	p__Bacteroidota; g__norank
OTU97	0.009966777	p__Bacteroidota; g__*Bacteroides*
OTU109	0.00877193	p__Firmicutes; g__[Eubacterium] ruminantium group
OTU121	0.007590133	p__Firmicutes; g__norank
OTU313	0.00729927	p__Firmicutes; g__Quinella
OTU76	0.005617978	p__Bacteroidota; g__norank

Normally distributed parametric data were analyzed by one-way ANOVA, followed by Brown-Forsythe and Welch’s post-hoc tests for multiple comparisons.

### Alpha diversity analysis

**FIGURE 4:**
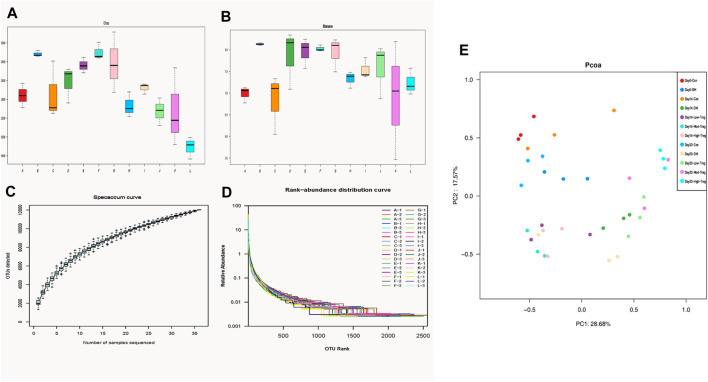
Diversity analysis: Chao index **(A)**, Shannon index **(B)**, the species specaccum curve **(C)**, the rank-abundance curve **(D)** and Beta diversity analysis **(E)**. **(A,B)** The larger the index, the higher the species richness. **(C)** abscissa: sample size; the ordinate: the number of OTUs after sampling. The results reflected the rate of emergence of new OTUs (new species) under continuous sampling. **(D)** In the horizontal direction, the abundance of species is reflected by the width of the curve. The higher the abundance of species, the larger the range of the curve on the horizontal axis. The shape of the curve (smoothness) reflects the evenness of the species in the sample. The gentler the curve, the more uniform the species distribution. **(E)** Based on Unweighted Unifrac distances, a PCoA analysis was conducted. PC1 and PC2 represent the suspected influencing factors for the shift of microbial composition in the two groups of samples, respectively.

In order to study the differences in gut microbiota diversity between T1DM mice and normal mice, we used Chao index, Shannon index, Simpson index to comprehensively characterize species diversity. The chaos index and shannon index of the control group on Day 0, 14, 30 were lower than the untreated diseased group (Figures 4A,B), indicating that the richness and diversity of microbes in the diseased group were greater than those in the normal group. At Day 30, the chaos index and shannon index of the untreated diseased group were higher than those of the Treg cell therapy group, and the index in the high-dose group was even lower, indicating that the richness and diversity of microbes in the therapy group were lower than those in the diseased group.

The species specaccum curve (Figure 4C) eflects the pattern of new OTU discovery within each individual group. For each group, before 25 samples are sequenced, the curve rises sharply, indicating that a large number of species in that group’s gut microbial community are continuously being discovered. As the sample size of the group increases further, the curve rises gradually, suggesting that new species in the group still appear but at a slower rate. By the time 35 samples are sequenced per group, the curve levels off, indicating that few new species are discovered with additional samples within that group. This suggests that the quantity of gut microbiota in mice tends towards a constant value with increasing sample size, showing that the sequencing data volume is reasonable and the sequencing depth is sufficient to reflect the community abundance in the samples. The rank-abundance curve (Figure 4D) demonstrates that the abundance of each species in every sample is clearly represented, with a relatively flat curve indicating a uniform distribution of species.

### Beta diversity analysis

Based on Unweighted Unifrac distances, a PCoA analysis was conducted (Figure 4E). The contribution of PC1 on the x-axis is 28.68%, while the contribution of PC2 on the y-axis is 17.57%. To quantify the differences between and within groups, Analysis of Similarities (ANOSIM) was performed, yielding a statistic R = 0.67 (*p* < 0.0010). This result confirms that differences between groups are greater than differences within groups, as delineated by time boundaries.

### Analysis of the screening of dominant microbial communities in each group

**FIGURE 5:**
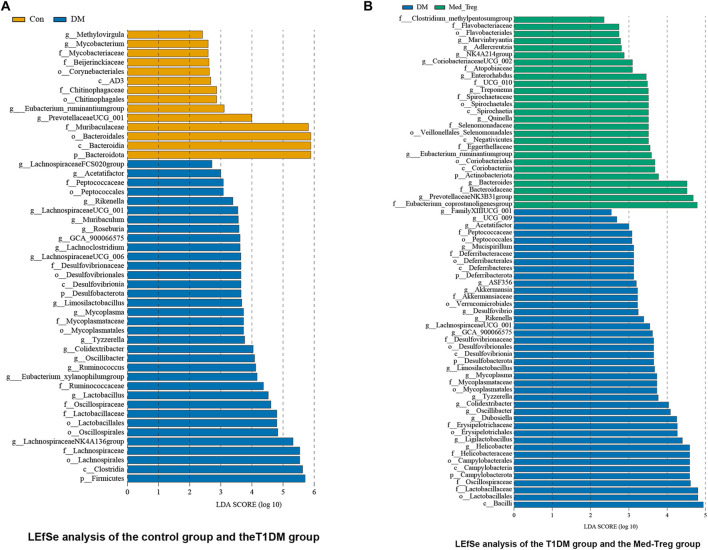
**(A)** LEfSe analysis of Day 14 samples, between the control group and the untreated T1DM group. **(B)** LEfSe analysis of Day 14 samples, between the untreated T1DM group and the medium-dose Treg treatment group.

In the animal experiments conducted in the early stage, it was found that on the 14th day, compared with the T1DM group, the mice in the high-dose treatment group showed relatively better improvement in fasting blood glucose, body weight, water intake, insulin, C-peptide and other indicators in Treg cells, with even better effects in the medium-dose group (Table 4 **)**. LEfSe analysis of Day 14 samples (Figure 5) revealed distinct microbial biomarkers. Compared to the control group, the untreated diseased group was enriched in the phyla Firmicutes and Desulfobacterota, and the genera Lachnospiraceae NK4A136 group, *Lactobacillus*, Eubacterium_xylanophilum_group, Ruminococcus, Oscillibacter, and Colidextribacter. In contrast, the medium-dose Treg treatment group showed a marked increase in the phylum Actinobacteriota and the genera Prevotellaceae NK3B31 group, Eubacterium_ruminantium_group, Quinella, *Treponema*, Enterorhabdus, and Corobacteriaceae UCG_002 compared to the untreated group.

**TABLE 4:** Blood glucose, body weight, water intake, insulin and C-peptide of mice in each group on Day14.Statistical analysis was performed using one-way ANOVA.

Item	Day14-Con	Day14-DM	Day14-low-treg	Day14-Med-treg	Day14-high-treg
Blood glucose (mmol/L)	5.72 ± 0.24	19.12 ± 0.52***	17.68 ± 0.09***	16.30 ± 0.50***#	15.85 ± 0.63***##
Weight(g)	21.78 ± 0.31	16.25 ± 0.20***	16.72 ± 0.49***	17.71 ± 0.23***	17.23 ± 0.41***#
Drinking amount (ml)	5.33 ± 0.33	27.17 ± 2.02***	23.17 ± 1.64***	23.33 ± 2.20***	20.67 ± 1.38***
Insulin (nIU/mL)	62.73 ± 4.47	19.47 ± 1.18***	20.05 ± 1.84***	21.78 ± 1.59***	28.94 ± 1.95***
C-peptide (ng/mL)	13.25 ± 0.71	6.27 ± 0.44***	6.42 ± 0.48***	7.80 ± 0.36***	8.50 ± 0.15***

Comparisons with the control group: *p < 0.0500, **p < 0.0100, ***p < 0.0010.

Comparisons with the T1DM, group: #*p* < 0.0500, ##*p* < 0.010.

Bold values indicate statistical significance at *p* < 0.05.

### Spearman correlation analysis of gut microbiota and immune biochemical expressions

**FIGURE 6:**
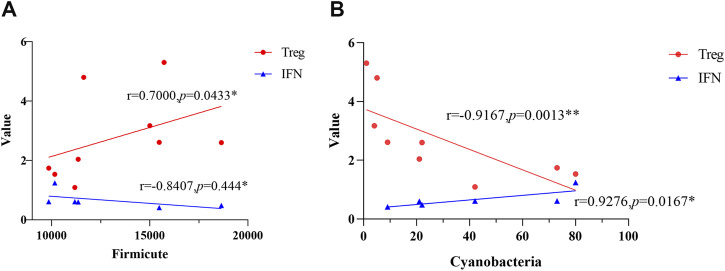
Spearman correlation analysis between top 10 abundant phyla and immune biochemical indicators. Pearson correlation coefficient *r* (−1 ∼+1) quantifies linear correlation: *r* = +1 (perfect positive linear correlation), *r* = 0 (no linear correlation), *r* = −1 (perfect negative linear correlation). Symbols * (*p* < 0.0500) and ** (*p* < 0.0100) indicate statistical significance of correlations. **(A)** Correlation between Firmicutes and immune indicators. Firmicutes was positively correlated with Treg levels (*r* = 0.70, *p* = 0.0433) and negatively correlated with IFN levels (*r* = −0.84, *p* = 0.4440). **(B)** Correlation between Cyanobacteria and immune indicators. Cyanobacteria was positively correlated with IFN levels (*r* = 0.9276, *p* = 0.0167) and negatively correlated with Treg levels (r = −0.9167, *p* = 0.0013).

Spearman correlation analysis was performed at the phylum level, initially including all 32 identified phyla. Results are presented for the 10 most abundant phyla, which collectively represented the majority of the microbial community. Spearman correlation analysis results showed a positive correlation between Firmicutes and Treg levels (*r* = 0.70, *p* = 0.0433) (Figure 6A), a negative correlation between Firmicutes and IFN (*r* = −0.84, *p* = 0.4440), a positive correlation between Cyanobacteria and IFN levels (*r* = 0.9276, *p* = 0.0167), and a negative correlation between Cyanobacteria and Treg levels (*r* = −0.9167, *p* = 0.0013) (Figure 6B). No statistically significant correlations were observed between the remaining phyla—Bacteroidota, Campylobacterota, Verrucomicrobiota, Proteobacteria, Actinobacteriota, Desulfobacterota, or Acidobacteriota—and any of the immune-biochemical markers assessed.

### PICTRUST function prediction

In comparison with the T1DM group on the 14th day, it was found in animal experiments that the improvement of indicators such as blood glucose and body weight in the Treg cell high-dose treatment group of mice was relatively better, and the medium-dose treatment group had even better effects (Table 2). Through reconstructing unobserved states for systematic phylogenetic research on the community using PICRUSt, gene prediction based on functional KEGG pathways was conducted on the sequenced genome to determine potential functional changes in the gut microbiota composition of mice in different groups on Day 14. Pathways involved in Fructose and mannose metabolism, Pentose phosphate pathway, Methane metabolism, Two-component system, ABC transporters, and others showed relatively higher abundance in the T1DM mouse group compared to the normal group, with statistically significant differences (*p* < 0.0500). Meanwhile, the normal group of mice showed higher relative abundance in pathways such as Oxidative phosphorylation, Arginine and proline metabolism, and Alanine, aspartate and glutamate metabolism compared to T1DM.

**FIGURE 7:**
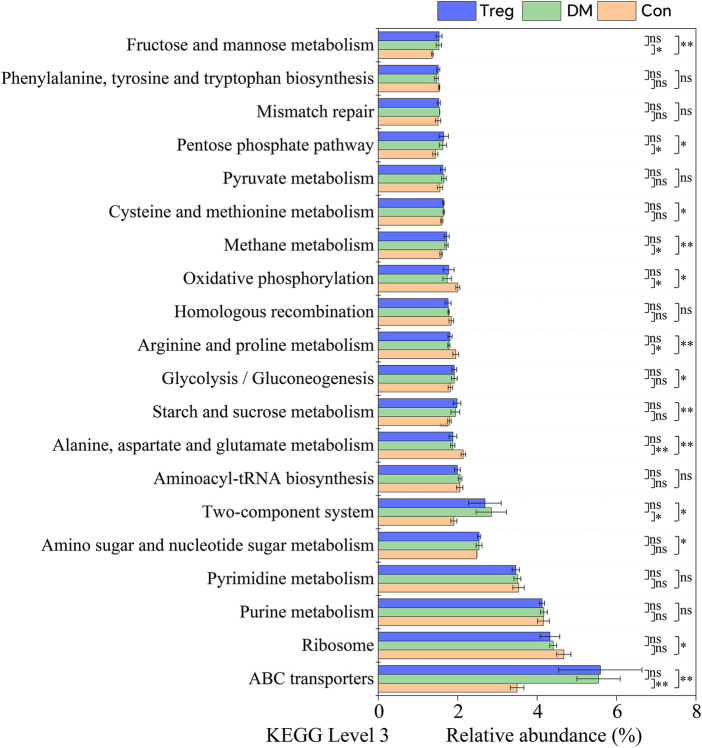
Comparative analysis of predicted KEGG pathway abundances in gut microbiota on Day 14. Functional prediction was performed using PICRUSt. Pathways including fructose and mannose metabolism, pentose phosphate pathway, methane metabolism, two-component system, and ABC transporters were significantly enriched in T1DM mice compared to normal controls (p < 0.0500). In contrast, pathways such as oxidative phosphorylation, arginine and proline metabolism, and alanine, aspartate and glutamate metabolism were more abundant in the normal group.

In addition to the pathways previously mentioned, the Treg cell treatment group showed higher relative abundances in several KEGG pathways compared to the normal control group, including fructose and mannose metabolism, pentose phosphate pathway, methane metabolism, two-component system, ABC transporters, cysteine and methionine metabolism, glycolysis/gluconeogenesis, and amino sugar and nucleotide sugar metabolism. Conversely, pathways such as oxidative phosphorylation, arginine and proline metabolism, alanine/aspartate/glutamate metabolism, and ribosome were less abundant in the Treg treatment group than in the normal group (Figure 7).

## Discussion

The gut microbiota supports host health by inhibiting pathogens, aiding mucosal repair, and maintaining immune balance. Accumulating evidence implicates it in the pathogenesis of type 1 diabetes (T1DM) [8]. Early work in germ-free non-obese diabetic (NOD) mice showed that pancreatic inflammation worsened in the absence of microbes but improved upon transfer to a conventional environment [9]. Subsequent studies found that disrupting the microbiota in NOD mice via long-term *Lactobacillus* acidophilus or antibiotic treatment led to intestinal barrier dysfunction, autoimmune reactions, aggravated pancreatic inflammation, and increased diabetes incidence. Conversely, probiotic intervention (e.g., VSL#3, containing bifidobacteria and lactobacilli) reduced diabetes incidence and preserved pancreatic β-cell mass [10]. These findings suggest that disrupting normal gut microbiota can induce or exacerbate T1DM. Consistently, early antibiotic treatment accelerates T1DM onset in NOD mice by altering the microbiota, metabolism, and T-cell populations [11], and dysbiosis-induced inflammation may worsen insulin resistance [12]. Conversely, drug interventions that regulate gut microbiota structure can improve pancreatic cell function, reduce the incidence of diabetes by balancing the microbiota structure [2]. Pharmacological modulation of the microbiota, such as with empagliflozin, can restore diversity, reduce lipopolysaccharide (LPS)-producing bacteria, increase short-chain fatty acid (SCFA) production, and improve diabetic outcomes by enhancing barrier function and reducing inflammation [13].

In line with this, our 16S rRNA sequencing revealed significant differences in α- and β-diversity among control, T1DM, and Treg cell-treated T1DM mice, indicating marked microbial alteration in T1DM that can be further modulated by therapy. Altered composition may contribute to pathogenesis, while Treg treatment may act by enriching beneficial taxa and reducing harmful ones, thereby restoring community structure. Interestingly, overall microbial richness was lower in Treg-treated mice than in untreated T1DM mice, a pattern differing from the partial diversity recovery often reported. This may reflect a pronounced suppression of pathogenic bacteria that is not fully offset by the expansion of beneficial taxa (e.g., Ruminococcaceae) within the 14-day intervention window. Although beneficial taxa were enriched post-treatment, their functional maturation (e.g., SCFA production) may require more time. Given the small sample size and exploratory design, we cannot establish causality but conclude that specific microbial shifts coincide with Treg administration and metabolic improvement in this model.

Based on heat maps and ANOVA analysis, we found that Verrucomicrobiota is significantly reduced in the diseased group compared to the normal group, suggesting that the decrease in Verrucomicrobiota is related to T1DM. Akkermansia is a representative genus of Verrucomicrobiota. Previous studies have found that Akkermansia can help reduce the risk of diabetes and inflammation [14]. Shi M and others found that it also can reduce LPS entering the bloodstream by protecting the intestinal mucus layer [15]. In addition, Akkermansia indirectly promotes the production of butyrate by producing high levels of acetate [16, 17]. The [Eubacterium] siraeum group was more abundant in T1DM mice at baseline, consistent with its reported negative correlation with glucose tolerance [18]. We observed a higher abundance of *Helicobacter* (phylum Campylobacterota) in the Day14-DM group compared to the Day14-Med-Treg group, with *H. pylori* being the most prevalent species. The relationship between *H. pylori* and diabetes is complex and may involve indirect effects on insulin resistance via metabolic and inflammatory pathways [19]. On the other hand, the hyperglycemic environment in diabetic patients can regulate immune cell function, stimulate pro-inflammatory cytokine production, leading to neurodegeneration, delayed gastric emptying, reduced gastric acid secretion, and increased risk of *H. pylori* infection [20, 21].

We observed a differential abundance of *Helicobacter* between T1DM and Treg-treated groups. This may be attributable to a direct modulatory effect of Treg cells on the gut microbiota or secondary to improved systemic glucose control. Notably, no significant difference in *Helicobacter* abundance was detected between T1DM and control groups at the genus level. This lack of difference could stem from the limited statistical power of our small sample size. Alternatively, it may reflect a weak or absent direct association between *H. pylori* and T1DM pathogenesis, as mechanistic links involving *H. pylori* (e.g., promotion of insulin resistance) are more established in type 2 diabetes [22]. Some scholars believe that in adolescents and children with T1DM, levels of glycated hemoglobin and poor blood sugar control are positively correlated with *H. pylori* infection [23]. Delitala and others found that highly toxic strains of *H. pylori* may induce immune-mediated diabetes [24]. In addition, alterations in the abundance of Tyzzerella, *Mycoplasma*, and the [Eubacterium] ruminantium group were observed in the T1DM group compared with the Medium-dose Treg group. Following Treg cell therapy, the abundance of Tyzzerella was reduced relative to the T1DM group. Given that Tyzzerella can produce substantial propionate—which downregulates cytokine-induced VCAM-1 expression and impairs insulin secretion [25, 26]—the decrease in Tyzzerella abundance induced by Treg cells may contribute to glycemic control through a concomitant increase in VCAM-1. *Mycoplasma*, commonly linked to respiratory infections, also colonizes the gastrointestinal tract. Previous studies indicate that Treg cells can modulate T-cell responses to *Mycoplasma* by enhancing IFN-*γ* and IL-17 production, thereby restraining destructive immune reactions in the respiratory tract [27]. The Gut–Lung Axis (GLA) theory posits a bidirectional interplay between intestinal and pulmonary immune systems and microbiota [28]. In the present study, *Mycoplasma* abundance was lower in the Treg-treated group than in the T1DM group, suggesting that Treg-mediated suppression of excessive immune responses may extend to intestinal *Mycoplasma* colonization. Moreover, through the GLA [29], Treg cells could indirectly regulate gut immune function via pulmonary immune modulation. Zhang et al. reported that Eubacterium promotes bladder cancer progression by upregulating extracellular matrix protein 1 (ECM1), which influences matrix metalloproteinase-9 (MMP9) expression in bladder tissue [30]. In tumor microenvironments, excessive Treg cell activation can inhibit anti-tumor immunity and facilitate “immune escape” [31]. Thus, the attenuated immune clearance associated with Treg cells in this study may potentially relate to changes in the [Eubacterium] ruminantium group.

Spearman correlation analysis indicated a positive correlation between Firmicutes abundance and Treg levels, and a negative correlation with IFN-γ. Firmicutes may influence host immune status and metabolic balance through metabolites such as short-chain fatty acids, which can modulate CD4^+^ T-cell differentiation [32] and thereby exert systemic effects. Treg cells are known to suppress IFN-γ secretion, reduce adipose tissue macrophage activation, and ameliorate insulin resistance [33]. Leite, A. Z and others observed a significant correlation between IFN-*γ* and the relative abundance of Firmicutes in patients with type 2 diabetes [34]. This could be attributed to Firmicutes-associated dysbiosis, which may increase intestinal permeability, promote LPS translocation, and activate the TLR4/NF-κB pathway, ultimately elevating inflammatory cytokines including IFN-γ and contributing to insulin resistance. In contrast, the Cyanobacteria phylum showed a negative correlation with Treg levels and a positive correlation with IFN-γ. Certain cyanobacteria such as spirulina can stimulate macrophages to produce IL-12, a cytokine that promotes IFN-γ synthesis by NK cells [35]. Additionally, cyanobacteria may downregulate IL-4, which normally inhibits spontaneous apoptosis of Tregs; reduced IL-4 could therefore enhance Treg cell death [36, 37]. Mesenchymal stem cells (MSCs) have been shown to suppress IFN-γ secretion by T and NK cells, attenuate adipose tissue macrophage activation, and promote Treg generation [38], thereby improving insulin sensitivity and supporting β-cell proliferation [39]. Consistent with this, earlier studies reported that gingival MSCs downregulated IL-17 and IFN-γ in CD4^+^CD8^+^ T cells, increased Treg numbers, and alleviated hyperglycemia and pancreatic inflammation in T1DM mice [5, 40]. In the present study, changes in C-peptide were used to assess pancreatic β-cell function. Overall, Firmicutes abundance correlated positively with C-peptide regulation, whereas Cyanobacteria exhibited a negative association.

Firmicutes are major producers of short-chain fatty acids (SCFAs) such as acetate, propionate, and butyrate, which contribute to gut barrier integrity, exert anti-inflammatory effects, and modulate glucose and lipid metabolism, blood pressure, and gut–brain axis signaling [41, 42]. On the other hand, Bifidobacterium—a key taxon linked to branched-chain amino acid biosynthesis and insulin resistance [18]—can also influence leptin signaling; its increased abundance may reduce energy intake and alter carbohydrate fermentation and lipopolysaccharide metabolism [43], thereby playing a beneficial role in glycemic control and T1DM prevention. The ratio of Firmicutes to Bacteroidetes (F/B ratio) is critical for polysaccharide fermentation. An elevated F/B ratio has been associated with inflammatory responses, increased body mass index, and diabetes development [44, 45]. In line with previous findings showing a higher duodenal F/B ratio in T1DM patients [46], our study observed an increased F/B ratio in the T1DM group compared with controls, while Treg treatment lowered this ratio. These results suggest that Treg cells may help restore microbial balance, improve metabolic regulation, and support weight management in T1DM.

Based on LEfSe analysis, the untreated disease group showed a distinct microbiota profile, characterized by dominance of the phyla Firmicutes and Desulfobacterota, and enrichment of the genera Lachnospiraceae NK4A136 group, *Lactobacillus*, Eubacterium_xylanophilum_group, Ruminococcus, Oscillibacter, and Colidextribacter. The increase in Firmicutes is consistent with reports linking a higher Firmicutes-to-Bacteroidetes ratio to a pro-inflammatory gut state. The rise in Desulfobacterota—a sulfate-reducing phylum—could further promote the production of hydrogen sulfide, a metabolite that impairs colonic epithelial cells and is associated with gut inflammation [47]. In support, Wei et al. observed a positive correlation between the relative abundance of Desulfobacterota and TNF-α levels [48]. In contrast, Treg cell therapy led to notable enrichment of several beneficial taxa. These included the short-chain fatty acid (SCFA)-producing genera Eubacterium_ruminantium_group and Quinella, as well as the family Atopobiaceae, which is regarded as potentially beneficial in the mammalian gut. Most Atopobiaceae species produce metabolites such as lactic acid and SCFAs, which help maintain intestinal homeostasis and attenuate inflammation by modulating Th17/Treg differentiation, autophagy, and inflammatory cell death pathways, thereby protecting against inflammatory diseases [49]. Additionally, the family Eggerthellaceae—which can convert ellagic acid into urolithins [50]—was enriched. Urolithins have been shown to mitigate high-fat-diet-induced weight gain via microbiota modulation [51], suggesting a possible role of Eggerthellaceae in improved weight regulation in our model. Collectively, these shifts indicate that Treg cells may promote metabolic benefits, at least in part, through the shaping of a more favorable gut microbiota composition.

To explore the functional consequences of gut microbiota alterations, we predicted KEGG pathways from 16S rRNA data and compared the metabolic profiles of the three groups at day 14. Compared with healthy controls, T1DM mice showed significant enrichment in pathways related to fructose and mannose metabolism, pentose phosphate pathway, methane metabolism, two-component system, and ABC transporters. This pattern corresponds to known metabolic dysregulation in T1DM. In line with this, intestinal methane producers have been reported to display impaired glucose tolerance under high carbohydrate loads and a greater tendency toward hyperglycemia [52], offering a plausible basis for the observed methane metabolism enrichment in T1DM mice. The two-component system has also been linked to diabetes pathology; for example, diabetes may promote nasal colonization of virulent *Staphylococcus* aureus—a common pathogen in diabetic foot infections—through modulation of this system [52]. ABC transporters were most enriched in the T1DM and Treg-treated groups, with levels significantly higher than in controls. ABC transporters are involved in various disease processes, including diabetes-related changes in bile formation due to altered renal tubular expression in STZ-induced diabetic rats [53, 54]. Within the immune system, ABC transporters—particularly ABCG1—contribute to Treg cell biology. Treg development depends on cholesterol homeostasis, where ABCG1-mediated cholesterol transport helps regulate intracellular cholesterol levels, thereby influencing Treg differentiation and function [55].

The limitations of our study should be acknowledged with emphasis on the exploratory nature of the current analyses, which is consistent with the pilot study design with n = 3 per group per time point. First, the small sample size inevitably compromises statistical power, increasing the randomness and uncertainty of the results. Second, as noted, the reliance on OTU-based clustering and PICRUSt predictive functional profiling further limits the robustness of taxonomic identification, correlation analyses, and KEGG pathway inferences. Thus, all conclusions derived from these analyses should be interpreted as exploratory rather than confirmatory. In addition, the lack of consistency in the timeframe of the study subjects does not rule out errors caused by individual differences in mice. It should also be clarified that the current results only represent changes in the gut microbiota of STZ-induced T1DM mice and cannot be directly generalized to clinical populations or other T1DM models. To validate and extend our observations, future studies should include larger cohorts and collect samples at multiple time points to delineate temporal dynamics in the gut microbiota. Such designs would help clarify whether the microbial shifts we observed are consistent and causally linked to disease progression or treatment response. Despite these limitations, our findings suggest that Treg cell therapy modifies the gut microbiota composition in STZ-induced T1DM mice. This supports the notion that the therapeutic effect of Treg cells may be partly mediated through the regulation of intestinal microbial communities.

## Data Availability

The datasets presented in this study can be found in online repositories. The names of the repository/repositories and accession number(s) can be found in the article/supplementary material.
